# Ceftriaxone‐induced liver injury: Incidence, phenotypic characterization and predictive factors

**DOI:** 10.1002/bcp.70657

**Published:** 2026-07-10

**Authors:** Mohamed Hatem, Mohannad N. AbuHaweeleh, Humam Emad Rajha, Mhd Osama Rahhal, Ahmad Hamdan, Lina Naseralallah

**Affiliations:** ^1^ Urology Department Hamad Medical Corporation Doha Qatar; ^2^ College of Medicine, QU Health Qatar University Doha Qatar; ^3^ Department on Internal Medicine Hamad Medical Corporation Doha Qatar; ^4^ Pharmacy Department, Hamad bin Khalifa Medical City Hamad Medical Corporation Doha Qatar

**Keywords:** ceftriaxone, cholestatic injury, drug‐induced liver injury, hepatotoxicity, incidence, predictors

## Abstract

**Introduction:**

Ceftriaxone is a widely used third‐generation cephalosporin in hospitalized patients. Although generally considered safe, it has been associated with hepatobiliary complications and emerging reports of drug‐induced liver injury (DILI). However, data on its true incidence, phenotypic patterns, and predictors remain limited. This study aimed to evaluate the incidence, characterize clinical phenotypes, and identify risk factors for ceftriaxone‐induced liver injury.

**Methods:**

A retrospective cohort study was conducted among adult hospitalized patients who received intravenous ceftriaxone between February 2023 and February 2024. DILI was defined according to predefined biochemical criteria, with additional low‐threshold criteria for early detection. Liver injury phenotypes were classified using the R ratio, and severity was graded according to Drug‐Induced Liver Injury Network (DILIN) criteria. Multivariable logistic regression was performed to identify independent predictors.

**Results:**

Among 739 patients, 244 (33.0%) developed low‐threshold liver test abnormalities, and 143 (19.4%) met biochemical criteria for DILI. Cholestatic injury was the predominant phenotype (46.2%), followed by hepatocellular (29.4%) and mixed patterns (24.5%). Mild and moderate abnormalities occurred in 24.1% and 14.9% of patients, respectively, whereas severe and life‐threatening abnormalities were observed in 7.2% and 7.0%. Longer treatment duration (≥6 days), proton pump inhibitor use, and higher baseline alkaline phosphatase were independently associated with increased odds of DILI.

**Conclusion:**

Ceftriaxone use was associated with a substantial burden of liver test abnormalities, including clinically meaningful elevations. Careful patient selection, limiting treatment duration, and routine liver monitoring may help mitigate DILI risk.

**What is already known about this subject**

- Ceftriaxone is a widely used third‐generation cephalosporin antibiotic for the treatment of serious bacterial infections in hospitalized patients; however, its broad antimicrobial spectrum, convenient once‐daily dosing and favourable safety profile have contributed to frequent empiric and sometimes inappropriate use in clinical practice.
- Ceftriaxone is generally considered relatively safe and is most commonly associated with mild adverse effects such as gastrointestinal symptoms, although more severe adverse events including neurotoxicity, hepatobiliary complications and drug‐induced liver injury (DILI) have also been reported.
- Although ceftriaxone‐associated DILI has historically been considered uncommon, increasing evidence suggests that clinically relevant hepatic injury may occur more frequently than previously recognized. Nevertheless, available data regarding the incidence, phenotypic characteristics and risk factors of ceftriaxone‐associated DILI remain limited and variable across studies.

**What this study adds**

- In this retrospective cohort of hospitalized patients, approximately one fifth met standardized biochemical criteria for DILI during ceftriaxone therapy, whereas one third developed low‐threshold liver biochemical abnormalities.
- Cholestatic injury was the predominant phenotype, although hepatocellular and mixed injury patterns were also observed.
- Longer ceftriaxone treatment duration, proton pump inhibitor use and higher baseline alkaline phosphatase levels were independently associated with increased odds of DILI.

## 1 INTRODUCTION

Ceftriaxone is a potent, third‐generation cephalosporin with excellent activity against many Gram‐negative and reasonable coverage of most Gram‐positive pathogens. Consequently, it is widely used for the treatment of diverse bacterial infections, including meningitis, bacteremia, pneumonia, urinary tract infections, skin and soft‐tissue infections, bone and joint infections, and gonorrhoea.^1^ Its prolonged half‐life allows for convenient once‐daily dosing and has contributed to its reputation as a safe, broad‐spectrum agent. However, these same characteristics have also facilitated its widespread—and often excessive—use as empiric therapy, frequently in the absence of clear clinical indications.^2^, ^3^

Multiple studies and prescribing audits consistently show substantial overuse and inappropriate utilization of ceftriaxone. For instance, an Ethiopian meta‐analysis revealed that approximately 55% of ceftriaxone prescriptions were inappropriate, either lacking clear justification or deviating from recommended dosing or indications.^3^ Similarly, a global study found that nearly half of ceftriaxone prescriptions were inappropriate overall, highlighting widespread deviation from guideline‐recommended use.^4^ Importantly, evidence from hospital‐based antimicrobial stewardship interventions—including prospective audit and feedback, guideline enforcement and post‐prescription review—indicates that nearly 30–50% of empiric ceftriaxone prescriptions are amenable to discontinuation, de‐escalation or substitution with narrower‐spectrum agents once clinical stability or microbiological data are available, without compromising patient outcomes.^5^ These findings highlight ceftriaxone as a major contributor to potentially avoidable broad‐spectrum antibiotic exposure and the subsequent acceleration of antimicrobial resistance.^6^, ^7^

Beyond its broader public health implications, ceftriaxone use is also associated with clinically relevant adverse effects. Milder and more commonly reported adverse effects include gastrointestinal symptoms such as diarrhoea, nausea and abdominal discomfort, as well as hypersensitivity reactions ranging from mild cutaneous eruptions to urticaria.^8^, ^9^ More serious, though less frequently recognized, adverse effects include central nervous system toxicity—manifesting as seizures, encephalopathy, confusion and hallucinations—particularly in older patients and those with renal impairment.^10^ In addition, ceftriaxone is associated with a spectrum of hepatic adverse effects, most notably hepatobiliary precipitation due to calcium–ceftriaxone complex formation, which may present as biliary sludge or pseudolithiasis and is typically reversible upon discontinuation.^11^ Although true drug‐induced liver injury (DILI) related to ceftriaxone has traditionally been regarded as rare, emerging reports suggest a broader array of clinical manifestations, ranging from asymptomatic elevations in liver enzymes to more severe hepatocellular, cholestatic or mixed patterns of injuries.^12^, ^13^ Nevertheless, data on the true incidence, severity, clinical phenotypes and predictors of ceftriaxone‐induced liver injury remain limited and inconsistent, precluding definitive conclusions regarding its clinical significance. A clearer understanding of the clinical patterns and risk factors associated with ceftriaxone‐induced liver injury is therefore essential to improve early recognition, guide monitoring strategies and optimize antimicrobial stewardship. Accordingly, this study aimed to evaluate the incidence of ceftriaxone‐induced liver injury, characterize its clinical patterns and severity and identify potential predictive factors associated with its development.

## 2 METHODS

### 2.1 Study design and patients

A retrospective cohort study was conducted at Hamad Medical Corporation (HMC), the largest tertiary care hospital in Qatar. Using HMC's electronic medical record system, we identified all adult patients (≥18 years) admitted to Hamad General Hospital and Ambulatory Care Center who received intravenous (IV) ceftriaxone between 1 February 2023 and 1 February 2024. Eligible patients were required to have both baseline and follow‐up liver function tests (LFTs) available during ceftriaxone therapy. Patients were excluded if they did not meet any of the criteria above or if critical data related to ceftriaxone dosing or treatment duration were missing.

A whole‐population (universal) sampling approach was employed, whereby all patients meeting the eligibility criteria during the study period were included; consequently, a formal sample size calculation was not performed.

### 2.2 Data collection

Four trained reviewers independently extracted data into a standardized Microsoft Excel data collection form. To ensure data accuracy, all records underwent dual verification by two senior authors, with discrepancies resolved through consensus. Collected variables included (1) patient demographics (age, sex and nationality); (2) ceftriaxone exposure characteristics (daily dose and total duration of therapy); (3) baseline comorbidities known to affect hepatic metabolism or liver function, including liver failure, diabetes mellitus, chronic kidney disease, malignancy and hepatitis B or C infection^14^, ^15^; (4) concomitant use of potentially hepatotoxic medications, including acetaminophen (>1500 mg/day for ≥3 consecutive days), proton pump inhibitors (PPIs), antiepileptic drugs (e.g., carbamazepine, levetiracetam, phenobarbital, phenytoin and zonisamide), hepatically metabolized antimicrobials (e.g., macrolides, clindamycin and metronidazole) and antituberculosis agents (e.g., isoniazid and rifampicin)^16^, ^17^; and (5) serial LFTs (ALT, AST, ALP and total bilirubin) obtained before, during and after ceftriaxone use.

### 2.3 Outcome definitions

Given the retrospective nature of this study, systematic and reliable assessment of clinical symptoms attributable to liver injury (e.g., fatigue, abdominal pain, pruritus or jaundice) was not consistently available across medical records. Accordingly, liver injury was assessed using biochemical criteria only, in line with established approaches in retrospective DILI research. DILI was defined biochemically according to the criteria proposed by Aithal et al.^18^ as the presence of any of the following: (1) alanine aminotransferase (ALT) or aspartate aminotransferase (AST) ≥ 5 times the upper limit of normal (ULN); (2) alkaline phosphatase (ALP) ≥ 2 times ULN; or (3) ALT ≥3 times ULN in conjunction with total bilirubin ≥2 times ULN.

In addition, a low‐threshold liver injury definition was applied to capture early or mild biochemical liver abnormalities that did not meet established DILI thresholds. This was defined as either (1) ALT ≥2 times ULN or (2) a concurrent increase in AST, ALP and total bilirubin, provided that at least one of these parameters exceeded two times ULN.^19^ This low‐threshold definition was employed for signal detection and characterization of early biochemical changes and was not considered diagnostic of DILI or indicative of clinical liver injury.

Patients meeting criteria for DILI were further evaluated to determine the liver injury phenotype using the R ratio,^18^, ^20^ a standardized biochemical tool that categorizes DILI based on the relative elevations of ALT and ALP. The *R* ratio was calculated as the ratio of ALT, expressed as a multiple of the ULN, to ALP, also expressed as a multiple of the ULN. Liver injury was subsequently categorized as hepatocellular (*R* ≥ 5), cholestatic (*R* ≤ 2) or mixed (2 < *R* < 5).^19^, ^21^ Use of the *R* ratio facilitates standardized phenotypic classification of liver injury and is widely recommended in DILI research to enhance consistency, comparability and interpretability across studies.

$$R\;\text{ratio}=\frac{\left(\mathrm{ALT}/{\mathrm{ULN}}_{\mathrm{ALT}}\right)}{\left(\mathrm{ALP}/{\mathrm{ULN}}_{\mathrm{ALP}}\right)}$$

The magnitude of liver enzyme abnormalities was further characterized using a Drug‐Induced Liver Injury Network (DILIN)‐based laboratory grading system, reflecting peak biochemical abnormalities. Liver injury was graded according to the DILIN biochemical severity criteria based on peak LFT values observed during follow‐up and classified as Grade 0 (normal), Grade 1 (mild), Grade 2 (moderate), Grade 3 (severe) or Grade 4 (life‐threatening).^21^, ^22^ Grading thresholds for ALT, AST, ALP and total bilirubin are summarized in Table 1. This grading system was applied to all patients exposed to ceftriaxone, including those who did not meet formal DILI criteria, to allow comprehensive characterization of the full spectrum of biochemical liver test abnormalities observed during therapy. Inclusion of all patients enabled assessment of both subclinical and clinically significant liver enzyme elevations, facilitating signal detection and comparison of laboratory abnormality patterns across exposure groups. Laboratory‐based grading provides an objective and standardized measure of the magnitude of liver enzyme abnormalities, enabling reproducible characterization and comparison of biochemical liver injury patterns across studies, independent of clinical symptom reporting. This approach is particularly relevant in retrospective studies, where systematic documentation of symptoms, jaundice and hepatic dysfunction may be incomplete.^21^, ^22^

**TABLE 1:** Severity grading of liver biochemical abnormalities according to DILIN criteria.

Feature	Grade 0	Grade 1	Grade 2	Grade 3	Grade 4
ALT	<1.25	1.25–2.5	>2.5–5.0	>5.0–10	>10
AST	<1.25	1.25–2.5	>2.5–5.0	>5.0–10	>10
Alkaline phosphatase	<1.25	1.25–2.5	>2.5–5.0	>5.0–10	>10
Bilirubin	Normal	>1.0–1.5	>1.5–2.5	>2.5–5	>5

*Note*: Grades were defined according to peak laboratory values expressed as multiples of the upper limit of normal (ULN).

Recovery was defined as normalization of all LFTs following ceftriaxone discontinuation.^22^, ^23^ Recovery status was assessed based on the first available post‐treatment LFTs obtained within <1 week, 1 month or 6 months following ceftriaxone discontinuation; cases without available follow‐up data were categorized as unavailable. The ULN values applied were ALT 40 U/L, AST 40 U/L, ALP 130 U/L and total bilirubin 21 μmol/L.

As liver enzyme abnormalities may also occur secondary to the underlying infection, causality assessment for suspected ceftriaxone‐induced liver injury was performed using the update RUCAM (Roussel Uclaf Causality Assessment Method); a validated and structured causality assessment tool for DILI was used for causality adjudication to determine the likelihood that observed liver injury was attributable to ceftriaxone.^20^ The RUCAM methodology assigns weighted scores across multiple domains, including time to onset of liver injury, course following drug discontinuation, risk factors, concomitant medications, exclusion of alternative causes, previous evidence of hepatotoxicity for the implicated agent and response to rechallenge where applicable. Causality assessment was independently conducted by two reviewers using the updated RUCAM scale. Discrepancies between reviewers were resolved through discussion and consensus, with involvement of a third reviewer where required to determine the final causality classification. Total scores were interpreted according to established classifications as highly probable (≥9), probable (6–8), possible (3–5), unlikely, (1–2) or excluded (≤0). The distribution of RUCAM causality categories among suspected cases is presented in Section 3 to provide additional context regarding attribution of liver injury to ceftriaxone. Detailed case‐level scoring analyses and further characterization of causality assessment findings will be reported separately.

### 2.4 Statistical analysis

Statistical analyses were performed using Stata/SE 19.5 (StataCorp LLC). Continuous variables were summarized for normality by histogram inspection. Normally distributed data were presented as means with standard deviations (SDs) and compared using Student's *t*‐test. Non‐normally distributed data were reported as medians and interquartile ranges (IQRs) and compared using the Wilcoxon rank‐sum test. Categorical variables were presented as frequencies and percentages (*n* [%]) and compared using Pearson's chi‐squared (χ^2^) test.

Among all cases, the magnitude of liver biochemical abnormalities was summarized descriptively. Among patients meeting DILI criteria, the distribution of liver injury phenotypes and recovery status were also summarized descriptively and presented in tabular format. To identify independent predictors of DILI, we fitted a multivariable logistic regression model adjusting for demographics, ceftriaxone exposure parameters (treatment duration was modelled categorically using ≤3 days as the reference), aforementioned comorbidities, concomitant hepatotoxic medications and baseline LFTs values. Results are reported as adjusted odds ratios (aORs) with 95% confidence intervals (CIs). Model diagnostics included specification testing using the link test, assessment of discriminatory performance using the receiver operating characteristic (ROC) curve and evaluation of multicollinearity using stata's *Linktest* command and Variance Inflation Factor (VIF). Statistical significance was defined as a two‐tailed *p* value < .05. All analyses were conducted using complete‐case data, given the minimal extent of missingness (<2%).

To evaluate the robustness of the findings and assess the potential impact of causality classification on identified predictors, a sensitivity analysis was performed using only cases classified as probable or highly probable by the RUCAM as the outcome. The multivariable logistic regression model was repeated using the same covariates as the primary analysis, and results were compared with those of the primary model.

### 2.5 Ethical considerations

The study was approved by the Institutional Review Board of HMC (MRC‐01‐23‐342) and conducted in accordance with the Declaration of Helsinki. Patient identifiers were removed prior to analysis to ensure their confidentiality. As this was a retrospective study using de‐identified data, investigators did not directly encounter or provide clinical care to individual study participants.

## 3 RESULTS

### 3.1 Baseline characteristics and clinical features stratified by DILI status

Baseline demographic and clinical characteristics of patients stratified by DILI status are presented in Table 2. Overall, these characteristics were generally comparable between patients who did and did not develop DILI. The mean age was similar between groups (57.4 ± 18.65 years *vs*. 56.3 ± 18.8 years), and males constituted a higher proportion of the DILI group (62.2% *vs*. 55.0%), although this difference was not statistically significant. Nationality distribution differed significantly between groups (*p* = .033); however, the overall regional pattern remained consistent, with patients from the Middle East comprising the largest proportion in both groups, followed by South Asia and North Africa, whereas other regions contributed smaller proportions.

**TABLE 2:** Baseline characteristics and clinical features stratified by DILI status.

Factor	Level	No DILI	DILI	*p* value
*N*		596	143	
Demographics				
Age, mean (SD)		56.31 (18.80)	57.40 (18.65)	.53
Gender, *n* (%)	Female	268 (45.0%)	54 (37.8%)	.12
	Male	328 (55.0%)	89 (62.2%)	
Nationality, *n* (%)	Middle East	337 (56.5%)	69 (48.3%)	.033
	South Asia	115 (19.3%)	27 (18.9%)	
	North Africa	84 (14.1%)	23 (16.1%)	
	Southeast Asia	34 (5.7%)	18 (12.6%)	
	Sub‐Saharan Africa	13 (2.2%)	4 (2.8%)	
	European and North American	12 (2.0%)	1 (0.7%)	
	East Asia	1 (0.2%)	0 (0.0%)	
	Latin America	0 (0.0%)	1 (0.7%)	
Baseline liver function tests & recovery				
Baseline total bilirubin, median (IQR)		7.00 (5.00, 12.00)	11.00 (7.00, 19.00)	<.001
Baseline ALP, median (IQR)		78.00 (63.00, 100.00)	91.00 (72.00, 130.00)	<.001
Baseline ALT, median (IQR)		17.00 (12.00, 25.00)	20.00 (12.90, 34.50)	.003
Baseline AST, median (IQR)		18.00 (14.00, 25.00)	21.00 (16.00, 37.00)	<.001
Comorbidities				
Liver failure, *n* (%)	No	571 (95.8%)	130 (90.9%)	.017
	Yes	25 (4.2%)	13 (9.1%)	
Diabetes mellitus, *n* (%)	No	345 (57.9%)	80 (55.9%)	.67
	Yes	251 (42.1%)	63 (44.1%)	
Chronic kidney disease, *n* (%)	No	485 (81.4%)	116 (81.1%)	.94
	Yes	111 (18.6%)	27 (18.9%)	
Malignancy, *n* (%)	No	504 (84.6%)	124 (86.7%)	.52
	Yes	92 (15.4%)	19 (13.3%)	
Hepatitis B or C virus, *n* (%)	No	578 (97.0%)	136 (95.1%)	.27
	Yes	18 (3.0%)	7 (4.9%)	
Medications				
Acetaminophen, *n* (%)^a^	No	542 (90.9%)	141 (98.6%)	.002
	Yes	54 (9.1%)	2 (1.4%)	
Proton‐pump inhibitors, *n* (%)	No	179 (30.0%)	21 (14.7%)	<.001
	Yes	417 (70.0%)	122 (85.3%)	
Antiepileptic drugs, *n* (%)	No	547 (91.8%)	132 (92.3%)	.84
	Yes	49 (8.2%)	11 (7.7%)	
Antimicrobial drugs, *n* (%)	No	277 (46.5%)	55 (38.5%)	.084
	Yes	319 (53.5%)	88 (61.5%)	
Antituberculosis drugs, *n* (%)	No	589 (98.8%)	142 (99.3%)	.62
	Yes	7 (1.2%)	1 (0.7%)	
Ceftriaxone use				
Duration of use (days), median (IQR)		4.50 (3.00, 7.00)	5.00 (4.00, 8.00)	<.001
Dose of ceftriaxone (gramme), *n* (%)	1	58 (9.7%)	11 (7.7%)	.47
	2	538 (90.3%)	131 (92.3%)	
Ceftriaxone duration (days), *n* (%)	3	168 (28.2%)	26 (18.2%)	.048
	4	130 (21.8%)	28 (19.6%)	
	5	86 (14.4%)	20 (14.0%)	
	6	57 (9.6%)	18 (12.6%)	
	7+	155 (26.0%)	51 (35.7%)	

*Note*: Data presented as mean (SD), median (IQR) or *n* (%). *p* values derived from two‐sample *t*‐test (continuous normally distributed variables); Wilcoxon rank‐sum test (continuous non‐normally distributed variables); Pearson's chi‐squared test (categorical variables). Institutional reference ranges were as follows: ALT, 0–41 U/L; AST, 0–40 U/L; alkaline phosphatase (ALP), 40–129 U/L; and total bilirubin, 0–21 μmol/L.

^a^ Acetaminophen use considered positive if >1500 mg/day for ≥3 consecutive days.

Patients who developed DILI had significantly higher baseline liver biochemical markers, including total bilirubin, ALP, ALT and AST (all *p* ≤ .003). Nevertheless, median baseline values in both groups remained within normal laboratory reference ranges. Baseline comorbidities were otherwise comparable; however, liver failure at baseline was more frequent among patients who developed DILI than among those who did not (9.1% *vs*. 4.2%, *p* = .017). The prevalence of diabetes mellitus, chronic kidney disease, malignancy and hepatitis B or C infection did not differ significantly between groups. Concomitant medication use varied between groups, with PPI use more common among patients who developed DILI (85.3% *vs*. 70.0%, *p* < .001), whereas acetaminophen use was less frequent (1.4% *vs*. 9.1%, *p* = .002).

Regarding ceftriaxone exposure, patients who developed DILI had a longer duration of therapy and were more likely to receive treatment for ≥7 days (*p* < .001), whereas ceftriaxone dosing did not differ significantly between groups.

### 3.2 Incidence of ceftriaxone‐induced liver injury

The occurrence of liver injury among patients receiving ceftriaxone is summarized in Table 3. Based on prespecified biochemical thresholds, low‐threshold liver injury was identified in 244 patients (33.0%). Using the biochemical criteria proposed by Aithal eh al.,^18^ 143 patients (19.4%) met formal diagnostic criteria for DILI. Thus, although biochemical liver test abnormalities were relatively common, a smaller subset fulfilled standardized DILI case definitions.

**TABLE 3:** Distribution of drug‐induced liver injury (DILI) phenotypes.

Factor	Level	Frequency	Percentage
Low‐threshold liver injury	Injury	244	33.02%
Drug‐induced liver injury, *n* (%)	DILI	143	19.35%
Type of liver injury, *n* (%)	Hepatocellular	42	29.40%
	Cholestatic	66	46.20%
	Mixed	35	24.50%
Recovered, *n* (%)^a^	No	84	58.70%
	Yes	59	41.30%

^a^ Recovered patients have their liver function tests return to normal after ceftriaxone use.

### 3.3 Causality assessment using RUCAM

Causality assessment was performed using the updated RUCAM scale among patients meeting biochemical criteria for DILI. Among suspected ceftriaxone‐induced liver injury cases, 5 patients (3.5%) were classified as highly probable, 54 (37.8%) as probable, 79 (55.2%) as possible, 3 (2.1%) as unlikely and 1 (0.7%) as excluded according to established RUCAM causality categories. These findings provide additional context regarding attribution of liver injury to ceftriaxone and support interpretation of the observed biochemical abnormalities. Detailed case‐level scoring analyses and characterization of individual causality domains will be reported separately.

### 3.4 Phenotypic distribution and recovery

The phenotypic distribution of liver injury among patients who developed DILI is summarized in Table 3. The most common pattern of liver injury was cholestatic (46.2%), followed by hepatocellular (29.4%) and mixed injury patterns (24.5%). Biochemical recovery following ceftriaxone exposure was observed in 41.3% of patients with DILI, whereas 58.7% did not achieve normalization of LFTs during the observation period. Among patients who achieved biochemical recovery, approximately 70% demonstrated normalization within 1 week of ceftriaxone discontinuation.

### 3.5 Magnitude of ceftriaxone‐induced liver injury

Peak LFT severity among patients receiving ceftriaxone is summarized in Table 4. The magnitude of peak LFT severity observed during ceftriaxone exposure is summarized in Table 4. Across the overall cohort, mild abnormalities occurred in 178 patients (24.1%), whereas moderate abnormalities were observed in 110 patients (14.9%). Severe and life‐threatening abnormalities were identified in 53 (7.2%) and 52 (7.0%) patients, respectively.

**TABLE 4:** Distribution of peak liver test abnormality severity across the study cohort.

Peak LFT level severity	Total frequency	Percentage	Low‐threshold liver injury	DILI
Normal	346	46.82%	0	0
Mild	178	24.09%	35	8
Moderate	110	14.88%	104	35
Severe	53	7.17%	53	51
Life threatening	52	7.04%	52	49

*Note*: Liver injury severity was graded based on the highest recorded values of alanine aminotransferase (ALT), aspartate aminotransferase (AST), alkaline phosphatase (ALP) and total bilirubin during the observation period. Grades were assigned according to the following criteria, using multiples of the upper limit of normal (ULN) for each parameter: Normal (Grade 0): all tests within normal limits (ALT < 1.25 × ULN, AST < 1.25 × ULN, ALP < 1.25 × ULN, Total Bilirubin ≤ 1.0 × ULN). Mild (Grade 1): at least one test at 1.25–2.5 × ULN for ALT/AST/ALP or >1.0–1.5 × ULN for bilirubin, with no test exceeding these ranges. Moderate (Grade 2): at least one test at >2.5–5 × ULN for ALT/AST/ALP or >1.5–2.5 × ULN for bilirubin, with no test exceeding these ranges. Severe (Grade 3): at least one test at >5–10 × ULN for ALT/AST/ALP or >2.5–5 × ULN for bilirubin, with no test exceeding these ranges. Life‐threatening (Grade 4): any test >10 × ULN for ALT/AST/ALP or >5 × ULN for bilirubin. The ULN values used were ALT = 40 U/L, AST = 40 U/L, ALP = 130 U/L, total bilirubin = 21 μmol/L.

### 3.6 Predictors of DILI

A multivariable logistic regression model was used to identify factors independently associated with the development of DILI during ceftriaxone therapy (Table 5). The model was statistically significant, suggesting the presence of at least one predictor of DILI.

**TABLE 5:** Multivariable logistic regression analysis of factors associated with DILI.

Factor	Level	Odds ratio	95% CI	Std. error	*p* value
Duration of use (days)	3 (control)	1.00 (ref)	—	—	—
	4	1.39	0.754–2.561	0.433	.291
	5	1.629	0.832–3.190	0.559	.154
	6	2.168	1.044–4.503	0.808	.038
	7+	2.277	1.302–3.983	0.65	.004
Dose category (gramme)	1	1.00 (ref)	—	—	—
	2	1.648	0.779–3.485	0.63	.191
Age group	Adult	1.00 (ref)	—	—	—
	Elderly	1.004	0.992–1.016	0.006	.559
Sex	Female	1.00 (ref)	—	—	—
	Male	1.358	0.900–2.049	0.285	.144
Liver failure	No	1.00 (ref)	—	—	—
	Yes	1.552	0.630–3.824	0.714	.34
Diabetes mellitus	No	1.00 (ref)	—	—	—
	Yes	1.037	0.662–1.625	0.238	.872
Chronic kidney disease	No	1.00 (ref)	—	—	—
	Yes	0.755	0.441–1.295	0.208	.308
Malignancy	No	1.00 (ref)	—	—	—
	Yes	0.766	0.419–1.398	0.235	.385
Hepatitis B/C	No	1.00 (ref)	—	—	—
	Yes	0.88	0.294–2.632	0.492	.819
acetaminophen use	No	1.00 (ref)	—	—	—
	Yes	0.115	0.026–0.499	0.086	.004
Proton pump inhibitor use	No	1.00 (ref)	—	—	—
	Yes	2.311	1.363–3.919	0.623	.002
Antiepileptic drug use	No	1.00 (ref)	—	—	—
	Yes	0.8	0.389–1.647	0.295	.545
Hepatic antimicrobial use	No	1.00 (ref)	—	—	—
	Yes	1.313	0.874–1.974	0.273	.19
Antituberculosis drug use	No	1.00 (ref)	—	—	—
	Yes	0.469	0.055–4.009	0.513	.489
Baseline ALP	Per unit	1.009	1.004–1.014	0.003	<.001
Baseline ALT	Per unit	1.001	0.987–1.015	0.007	.901
Baseline AST	Per unit	1.011	0.996–1.025	0.008	.16
Constant		0.01	0.003–0.037	0.007	<.001

*Note*: Results from a single multivariable logistic regression model (*N* = 738) with drug‐induced liver injury (DILI) as the outcome. All variables were included in a multivariable logistic regression model predicting DILI. The model is adjusted for dose category, elderly status, sex, liver failure, diabetes mellitus, chronic kidney disease, malignancy, hepatitis B or C virus infection, concomitant use of acetaminophen, proton‐pump inhibitors, antiepileptic drugs, liver‐metabolized antimicrobials and antituberculosis drugs, as well as pre‐treatment levels of ALP, ALT and AST. The reference group for duration of treatment was defined as patients treated with ceftriaxone for 3 days or less (control group). All categorical variables are presented with their respective reference levels as indicated in the table. Odds ratios (ORs) > 1 indicate increased odds of DILI, whereas ORs < 1 indicate decreased odds. *p* values are two‐tailed; statistical significance set at *α* = .05. Confidence intervals (CI) are reported as [lower limit − upper limit].

Abbreviation: Ref, reference category.

After adjustment for all covariates, longer duration of ceftriaxone therapy was independently associated with increased odds of DILI. Compared with treatment durations of ≤3 days (reference), therapy lasting 6 days (OR 2.17, 95% CI 1.04–4.50, *p* = .038) and ≥7 days (OR 2.28, 95% CI 1.30–3.98, *p* = .004) was associated with significantly higher risk of DILI, whereas treatment durations of 4 and 5 days were not statistically significant.

PPI use was independently associated with increased odds of DILI (OR 2.31, 95% CI 1.36–3.92, *p* = .002), whereas acetaminophen use was associated with reduced odds of DILI (OR 0.12, 95% CI 0.03–0.50, *p* = .004). Higher baseline ALP levels were also independently associated with DILI risk (OR 1.009 per unit increase, 95% CI 1.004–1.014, *p* < .001).

No statistically significant associations were observed for ceftriaxone dose, age group, sex, liver failure, diabetes mellitus, chronic kidney disease, malignancy, hepatitis B or C infection or concomitant use of antiepileptic, hepatic antimicrobial or antituberculosis drugs.

A sensitivity analysis restricted to cases classified as probable or highly probable by RUCAM was performed to evaluate the robustness of the identified predictors (Table S1). The findings were largely consistent with the primary analysis, with treatment duration ≥7 days (OR 3.71, 95% CI 1.61–8.51; *p* = .002), PPI use (OR 2.73, 95% CI 1.26–5.92; *p* = .011) and higher baseline ALP levels (OR 1.017 per unit increase, 95% CI 1.009–1.025; *p* < .001) remaining independently associated with DILI. In addition, increasing age was independently associated with DILI risk in the sensitivity analysis (OR 1.024 per year, 95% CI 1.006–1.043; *p* = .007).

## 4 DISCUSSION

### 4.1 Principal findings

In this cohort of patients receiving ceftriaxone, liver test abnormalities were common, with approximately one third of patients developing low‐threshold liver injury signals and nearly one fifth fulfilling the biochemical criteria for DILI proposed by Aithal et al.^18^ Among suspected cases, RUCAM causality assessment demonstrated that approximately 41% were classified as probable or highly probable, whereas 55.2% were categorized as possible. Among affected patients, cholestatic injury represented the predominant phenotype (46.2%). The spectrum of peak liver test abnormalities showed that mild and moderate abnormalities were observed in 178 (24.1%) and 110 (14.9%) patients, respectively; although less frequent, severe or life‐threatening biochemical derangements still occurred in 105 patients (14.2%) of the cohort. In multivariable analysis, longer duration of ceftriaxone therapy, PPI use and higher baseline ALP levels were independently associated with increased odds of DILI.

### 4.2 Incidence of DILI

The present study identified that 19.4% of patients met the biochemical criteria proposed by Aithal et al.,^18^ which exceeds the range reported in prior observational studies (2.9–13.9%).^24^, ^25^, ^26^ Among suspected cases, RUCAM causality assessment classified 41.3% as probable or highly probable and 55.2% as possible, providing additional context regarding attribution of liver injury to ceftriaxone. However, this estimate is comparable to incidence rates observed in cohorts receiving ceftriaxone alongside other potentially hepatotoxic agents.^12^ Differences in study design and patient population likely contribute to this variability across studies.

Unlike many previous investigations conducted in mixed inpatient–outpatient or community‐based cohorts, our study included exclusively hospitalized patients, including individuals with critical illness and substantial comorbidity burden. Such populations are recognized to be at increased risk for hepatotoxicity due to polypharmacy, systemic inflammatory states and altered drug metabolism, factors that may collectively increase susceptibility to antibiotic‐associated liver injury.^27^, ^28^

In addition, our study employed a two‐tiered detection framework, capturing not only cases meeting the biochemical criteria for DILI proposed by Aithal et al.^18^ but also a broader category of low‐threshold biochemical abnormalities representing potential hepatotoxicity signals. This approach revealed that liver test abnormalities occurred in approximately one third of patients, suggesting that subclinical hepatic perturbations during ceftriaxone therapy may be more common than previously appreciated. Furthermore, variability in DILI definitions across studies complicates direct comparison, as differing biochemical thresholds and case adjudication methods can substantially influence reported incidence estimates.^29^ These observations underscore the importance of standardized definitions and systematic monitoring strategies when evaluating antibiotic‐associated liver injury.

### 4.3 Phenotypic pattern of DILI

In our study, cholestatic injury represented the predominant phenotype of ceftriaxone‐associated DILI, accounting for 46.2% of cases. This finding aligns with the hepatobiliary profile reported for other cephalosporins as well as several other antibiotics agents commonly associated with cholestatic liver injury.^30^ Reports of ceftriaxone‐associated biliary sludge formation, pseudolithiasis and reversible cholestasis may support this observed predominance.^11^

Despite the predominance of cholestatic injury, hepatocellular and mixed patterns were also observed, highlighting the heterogeneous nature of ceftriaxone‐associated liver injury. Variability in injury phenotype may reflect differences in patient characteristics, concomitant medications and underlying clinical conditions. These findings suggest that liver injury during ceftriaxone therapy may present with diverse biochemical patterns and support comprehensive monitoring of both hepatocellular and cholestatic liver parameters during treatment.

### 4.4 Magnitude of DILI

Evaluation of peak liver test abnormalities demonstrated a broad spectrum of hepatic injury during ceftriaxone exposure. Although most patients experienced mild to moderate abnormalities, approximately 14% developed severe or life‐threatening biochemical derangements. These findings suggest that ceftriaxone‐associated liver injury may extend beyond minor laboratory abnormalities in a subset of patients. However, interpretation should be cautious, as severity grading was based on biochemical abnormalities without systematic clinical correlation, which is an inherent limitation of retrospective analyses.

### 4.5 Risk factors of DILI

Duration of ceftriaxone therapy emerged as a strong independent predictor of DILI, with a graded increase in risk observed across treatment duration categories and statistically significant associations emerging at longer durations (≥6 days), supporting a temporal exposure–response relationship. Although ceftriaxone dose was not identified as a significant risk factor, this finding likely reflects the limited variability in dosing within the cohort, with most patients receiving 2 g once daily and only a small proportion receiving 1 g. Although higher total daily exposure through more frequent dosing is used in clinical practice, such as ceftriaxone 2 g twice daily for bacterial meningitis among other indications, this regimen was not observed in our study population. Therefore, the absence of a dose effect should not be interpreted as evidence against an exposure–response relationship, as cumulative exposure duration remained a robust determinant of DILI risk. Prolonged cephalosporin exposure may allow greater accumulation of drug–protein adducts, promote adaptive immune activation or exceed individual thresholds for immune‐mediated hepatic injury, thereby increasing the likelihood of clinically manifest hepatotoxicity.

Baseline liver biochemical parameters, including AST, ALT, ALP and bilirubin, were associated with increased DILI risk, despite values remaining within normal laboratory ranges across groups. This observation suggests that subtle variations in baseline hepatic function may reflect underlying vulnerability to hepatotoxic injury, even in the absence of overt biochemical abnormalities. Such findings are consistent with the concept that reduced hepatic reserve or subclinical hepatic alterations may predispose patients to DILI.^31^

Older age (≥65 years) was also associated with increased DILI risk, highlighting the potential contribution of age‐related physiologic changes, including reduced hepatic mass and perfusion, diminished synthetic capacity and altered drug metabolism.^32^ These factors may collectively increase susceptibility to hepatotoxic effects in elderly populations and have been implicated in prior studies evaluating age‐related DILI risk.^32^, ^33^

Among concomitant medications evaluated, PPI use was independently associated with increased odds of DILI, whereas other potentially hepatotoxic agents, including antiepileptic drugs, hepatically metabolized antimicrobials and antituberculosis medications, did not demonstrate significant associations. This differential finding may partly reflect variation in injury phenotype, as several evaluated medications are more commonly associated with hepatocellular injury, whereas PPIs have been linked, albeit rarely, to cholestatic or mixed patterns that may align more closely with ceftriaxone‐associated hepatobiliary effects.^34^ In addition, the high prevalence of PPI exposure in hospitalized populations, particularly among critically ill patients receiving stress ulcer prophylaxis,^35^ may have increased the statistical power to detect an association relative to less frequently used agents. In contrast, acetaminophen exposure appeared to be associated with reduced odds of DILI; however, this finding should be interpreted cautiously, as only two patients in the DILI group met exposure criteria (>1500 mg/day for ≥3 consecutive days), indicating limited use within this subgroup and suggesting that the observed association likely reflects sparse exposure rather than a true protective effect.

Importantly, the associations between prolonged ceftriaxone exposure, PPI use and higher baseline ALP levels remained largely unchanged in a sensitivity analysis restricted to cases classified as probable or highly probable by RUCAM, supporting the robustness of the identified predictors. In this sensitivity analysis, increasing age was also independently associated with DILI risk, further suggesting that older patients may represent a particularly vulnerable population for ceftriaxone‐associated liver injury.

### 4.6 Clinical implications and management recommendations

The findings of this study suggest that ceftriaxone use in hospitalized patients may be associated with a higher burden of DILI than previously reported, highlighting the importance of vigilant hepatic monitoring during therapy, particularly given that a subset of cases demonstrated severe biochemical abnormalities. Particular attention may be warranted in patients with baseline hepatic vulnerability, advanced age, prolonged treatment duration and concurrent PPI exposure. Given the predominance of cholestatic injury patterns and the observed spectrum of biochemical abnormalities, routine monitoring strategies that incorporate both aminotransferases and cholestatic markers may facilitate early detection of evolving hepatic injury.

These findings also underscore the importance of judicious ceftriaxone prescribing within antimicrobial stewardship frameworks, ensuring that its use remains appropriately indicated and duration is carefully considered. Collectively, these observations support a pragmatic approach to ceftriaxone therapy that balances its favourable antimicrobial profile with targeted biochemical surveillance in higher‐risk populations.

### 4.7 Strengths and limitations

This study has several notable strengths. The use of a dual detection framework incorporating both proposed by Aithal et al.^18^ and a low‐threshold biochemical definition allowed comprehensive characterization of ceftriaxone‐associated hepatic effects, facilitating identification of both clinically defined DILI and early hepatotoxicity signals. In addition, liver injury was assessed using a structured and comprehensive approach consistent with contemporary recommendations, including phenotypic classification based on the R ratio, grading of injury magnitude and causality adjudication using the updated RUCAM methodology.^18^, ^36^ Furthermore, the inclusion of a large hospitalized cohort and detailed evaluation of demographic, clinical and pharmacologic variables enabled exploration of multiple potential risk factors for ceftriaxone‐associated liver injury. Sensitivity analyses restricted to probable and highly probable RUCAM cases yielded findings that were largely consistent with the primary analysis, further supporting the robustness of the identified predictors.

Several limitations should also be acknowledged. First, the retrospective design introduces inherent constraints related to data completeness and accuracy, including potential misclassification of exposure and outcome variables. The precise timing of DILI onset, presence of clinical symptoms and assessment of potential confounding factors may have been incompletely captured in medical records. Although vasopressor use was evaluated as a marker of hemodynamic instability, ischemic or sepsis‐related hepatic injury could not be fully excluded in all patients. Prospective studies with standardized data collection would strengthen causal inference and improve severity characterization. Second, biochemical criteria were used to define liver injury in this study, and the magnitude classification relied on laboratory abnormalities without systematic clinical correlation. Consequently, the clinical severity of observed biochemical derangements could not be fully established, and laboratory‐based grading may not completely reflect patient‐level clinical impact. Third, although causality assessment was strengthened through application of the updated RUCAM methodology, most suspected cases were classified as possible rather than probable or highly probable, reflecting the inherent challenges of causality attribution in hospitalized patients with competing causes of liver biochemical abnormalities. Finally, heterogeneity in DILI definitions across studies complicates direct comparison of incidence estimates. Although the biochemical criteria proposed by Aithal et al.^18^ were applied, differences in thresholds, adjudication methods and monitoring practices may influence reported incidence rates. Greater standardization of international consensus definitions would enhance comparability across future studies.

## 5 CONCLUSION

In this cohort of hospitalized patients, ceftriaxone use was associated with a substantial incidence of DILI, predominantly cholestatic in pattern. Although most liver test abnormalities were mild to moderate, a notable subset of patients developed severe or life‐threatening biochemical elevations. PPI use, higher baseline ALP and prolonged treatment duration, were independently associated with increased risk, with treatment duration supporting a temporal exposure–response relationship, and were also linked to greater odds of DILI. These findings highlight the importance of appropriate indication, careful limitation of treatment duration and routine hepatic monitoring during ceftriaxone therapy.

## CONFLICT OF INTEREST STATEMENT

The authors declare no conflicts of interest.

## Supporting information


**Table S1** Sensitivity analysis of predictors of drug‐induced liver injury restricted to probable and highly probable RUCAM cases.

## Data Availability

No additional datasets were generated or analysed during the current study.
