# Supplementary material for: Ceftriaxone‐induced liver injury: Incidence, phenotypic characterization and predictive factors
Source: Br J Clin Pharmacol. 2026 Jul 10;92(11):3874–85. doi: 10.1002/bcp.70657 (PMC13714625; doi:10.1002/bcp.70657)
Supplement: Supplementary file 1 [file BCP-92-3874-s001.docx]

**Supplementary Table S1. Sensitivity analysis of predictors of drug-induced liver injury restricted to probable and highly probable RUCAM cases**

| Factor | Level | Odds Ratio | 95% CI | Std. Error | p-value |
| --- | --- | --- | --- | --- | --- |
| **Duration of Use (days)** | 3 (Control) | 1.00 (ref) | — | — | — |
|  | 4 | 1.204 | 0.434–3.344 | 0.627 | 0.722 |
|  | 5 | 1.242 | 0.410–3.769 | 0.703 | 0.701 |
|  | 6 | 2.644 | 0.893–7.831 | 1.465 | 0.079 |
|  | 7+ | **3.705** | 1.613–8.512 | 1.572 | **0.002** |
| **Dose Category (gram)** | 1 | 1.00 (ref) | — | — | — |
|  | 2 | 2.295 | 0.637–8.274 | 1.502 | 0.204 |
| **Liver Failure** | No | 1.00 (ref) | — | — | — |
|  | Yes | 1.00 (empty) | — | — | — |
| **Diabetes Mellitus** | No | 1.00 (ref) | — | — | — |
|  | Yes | 0.677 | 0.352–1.302 | 0.226 | 0.243 |
| **Chronic Kidney Disease** | No | 1.00 (ref) | — | — | — |
|  | Yes | 0.574 | 0.258–1.277 | 0.234 | 0.174 |
| **Malignancy** | No | 1.00 (ref) | — | — | — |
|  | Yes | 0.765 | 0.313–1.869 | 0.349 | 0.557 |
| **Hepatitis B/C** | No | 1.00 (ref) | — | — | — |
|  | Yes | 1.00 (empty) | — | — | — |
| **Acetaminophen Use** | No | 1.00 (ref) | — | — | — |
|  | Yes | 1.00 (empty) | — | — | — |
| **Proton Pump Inhibitor Use** | No | 1.00 (ref) | — | — | — |
|  | Yes | **2.732** | 1.262–5.916 | 1.077 | **0.011** |
| **Antiepileptic Drug Use** | No | 1.00 (ref) | — | — | — |
|  | Yes | 0.142 | 0.019–1.076 | 0.147 | 0.059 |
| **Hepatic Antimicrobial Use** | No | 1.00 (ref) | — | — | — |
|  | Yes | 0.633 | 0.351–1.143 | 0.191 | 0.129 |
| **Antituberculosis Drug Use** | No | 1.00 (ref) | — | — | — |
|  | Yes | 0.924 | 0.093–9.226 | 1.085 | 0.946 |
| **Sex** | Female | 1.00 (ref) | — | — | — |
|  | Male | 1.206 | 0.659–2.204 | 0.371 | 0.543 |
| **Age** | per year | **1.024** | 1.006–1.043 | 0.009 | **0.007** |
| **Baseline ALP** | per unit | **1.017** | 1.009–1.025 | 0.004 | **<0.001** |
| **Baseline ALT** | per unit | 0.999 | 0.976–1.022 | 0.012 | 0.926 |
| **Baseline AST** | per unit | 0.993 | 0.963–1.024 | 0.015 | 0.660 |
| **Constant** |  | 0.0013 | 0.00016–0.0101 | 0.0014 | **<0.001** |
